# Specific Marker Gene Analysis for Primary Central Nervous System Lymphoma Based on Methylation Difference and Development of Detection Primers

**DOI:** 10.1002/brb3.70996

**Published:** 2025-10-15

**Authors:** Feng Yan, Yaming Wang, Xiaotong Fan, Penghu Wei, Yongzhi Shan

**Affiliations:** ^1^ Department of Neurosurgery Xuanwu Hospital Capital Medical University Beijing China; ^2^ China International Neuroscience Institute (China‐INI) Beijing China; ^3^ China National Medical Center for Neurological Diseases Beijing China

**Keywords:** 450 K microarray analysis, methylation, primary central nervous system lymphoma, RRBS analysis

## Abstract

**Background:**

Primary central nervous system lymphoma (PCNSL) is a central nervous system disease with high mortality, wide variation in symptoms, and difficult diagnosis. The development of molecular markers for PCNSL is still in its initial stages.

**Methods:**

The databases were analyzed by 450 K and RRBS, and the differential gene structure was displayed through Venn diagram, heatmap, and enrichment. ROC and survival curve analyses were performed on the marker genes. The specificity of PCNSL in blood samples of patients was verified by qPCR, and the sensitivity of the detection primers was verified by in vitro methylation and demethylation drug treatment cell experiments.

**Results:**

Twenty‐six sites were identified using 450 K microarray analysis. The RRBS analysis team found 14,867 sites. The methylation site located in the promoter region of REHB was verified, and the results showed that the primers for this site were able to distinguish patients with PCNSL and were sensitive to the detection of methylation levels.

**Conclusions:**

A pair of methylation primers targeting the RHEB promoter region were obtained, which demonstrated the potential to distinguish patients with PCNSL from those with other CNS diseases. These findings should be considered preliminary and serve as proof‐of‐concept for further validation in larger cohorts.

## Graphical abstract

Promoter hypermethylation of the RHEB gene is identified as a specific biomarker for Primary Central Nervous System Lymphoma (PCNSL). This study develops methylation‐specific primers, demonstrating their power to distinguish PCNSL from other CNS diseases via a novel plasma‐based detection method.

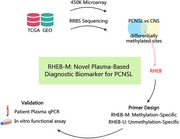

## 1 Introduction

Central nervous system (CNS) damage can result from a variety of causes, among which primary CNS lymphoma (PCNSL) is an extratodular non‐Hodgkin lymphoma of the brain, spine, cerebrospinal fluid, or vitreoretinal space (Grommes and DeAngelis 2017; Grommes 2020). PCNSL is a rare cancer with low incidence; however, its risk increases with age (Mendez et al. 2018; Lv et al. 2022). PCNSL accounts for 4%–6% of all extranodal lymphomas and approximately 4% of malignant brain tumors (Villano et al. 2011). PCNSL can occur at any age, but the median age is 56–61 years; 0.4% occurs in patients younger than 9 years, 1.5% occurs in patients younger than 19 years (Mendez et al. 2018), and the risk of PCNSL is greatly increased in immunosuppressed patients. Some patients showing exteroceptive neurological deficits, some patients showing non‐specific cognitive or behavioral changes, and signs of elevated intracranial pressure also appear in patients (Bataille et al. 2000); however, these disorders are not easily identified as PCNSL. Most patients with PCNSL and eye involvement have no visual symptoms (Grimm et al. 2007). Therefore, PCNSL is often misdiagnosed. Approximately 20% of patients with PCNSL may have pial involvement at the time of diagnosis; therefore, staging can be achieved using lumbar puncture. As the diagnosis of PCNSL is difficult, its detection is complicated, and the diagnosis time needs to be controlled, it is necessary to develop more convenient diagnostic markers. The use of disease‐specific circulating biomarkers can avoid complex detection processes, and it is feasible to use sequencing technology to develop PCNSL disease markers, including protein markers or small RNA (Grommes et al. 2019; Duan et al. 2015). However, the specificity of these markers is often limited, and the effect is not good (Baraniskin and Schroers 2014). In recent years, the development of DNA methylation markers has shown that methylation marker primers can be widely used for tumor diagnosis, treatment, and prognosis (Downs et al. 2019; Fiano et al. 2014), the development of PCNSL‐specific methylation markers is still in its infancy, with only a few reports (Richter et al. 2009; Vogt et al. 2019). It focused on non‐CNS diffuse large B‐cell lymphoma and did not consider other CNS tumors. Therefore, 450 K methylation site analysis based on gene chip analysis and RRBS methylation site analysis based on whole‐genome scanning were used in this study. TCGA, National Cancer Institute (NCI) Genomic Data Commons, CCLE Browse Datasets, and the Gene Expression Omnibus GSE data downloaded from the database were analyzed using PCNSL‐specific markers, and the ability of markers to distinguish the plasma of PCNSL patients from other CNS diseases was verified by clinical data to obtain clinically available PCNSL‐specific molecular markers.

## 2 Materials and Methods

### 2.1 Data Sources

All the processing K450 chip analysis data were collected from TCGA (Human Methylation 450K) and were downloaded from the NCI Genomic Data Commons (https://gdc.cancer.gov/), including diffuse large B‐cell lymphoma (DLBCL, *N* = 48), glioblastoma and lower grade gliomas (GBM and LGG, *N* = 685), Pheochromocytoma & Paraganglioma (PCPG, *N* = 187) and Colon Cancer (COAD, *N* = 100). All the processing RRBS data were derives from CCLE Browse Datasets (https://portals.broadinstitute.org/ccle/data) and the Gene Expression Omnibus database (http://www.ncbi.nlm.nih.gov/geo/).

The CNS tumors data were accessible under GSE70175 and GSE27584. The normal tissue data are accessible under GSE42590, GSE45341, GSE50852, GSE55249, GSE70175, GSE97106, GSE112524, GSE27584, and GSE17312. The DLBCL data are accessible under GSE66329.

### 2.2 Data Processing

We obtained mHap files with mHapSuite (https://github.com/yoyoong/mHapSuite), mean methylation (MM), PDR, CHALM, MCR, MBS, MHL, entropy, and linkage disequilibrium (LD) *R*‐squared (*R* ^2^). The analysis focused on gene promoters, defined as 1500 bp regions centered at the transcription start site (TSS).

### 2.3 Differential Methylation Analysis

We first merged all the samples by group based on the methylated gene promoters and performed differential methylation analysis by comparing two groups of samples using Student's *t*‐test. Significantly differentially methylated gene promoters were determined with a resulting *p*‐value cutoff of 5% plus absolute changes in MM of 0.1.

### 2.4 Enrichment Analysis

Additionally, we applied enrichment analyses, including Gene Ontology (GO) and the Kyoto Encyclopedia of Genes and Genomes (KEGG) pathway for differentially methylated sites. The most enriched pathways were visualized using ClusterProfiler.

### 2.5 Overlap With 450k Methylation Data

The bed files containing methylated sites were analyzed using GenomicFeatures (https://github.com/Bioconductor/GenomicFeatures) to identify the common methylated sites in the RRBS and 450k methylation data, as well as the regions with relevant genomic features.

### 2.6 Marker Identification

DNA methylation levels for the CpGs data are reported as *β* values, which is defined as the ratio of the intensity of the methylated allele to the overall intensity. The CpG sites that had a *β* value less than 0.15 in non‐CNS tumors, ranging from 0.2 to 0.3 in CNS tumors and greater than 0.4 in PCNSL in the mean time were selected using this program.

### 2.7 Marker Verification

To test the ability of the markers to distinguish PCNSL, the area under the curve (AUC) and 95% CI for each marker were calculated using abovementioned GEO datasets, the ROC curve was done using “sklearn” module in Python as well.

### 2.8 Survival Analysis

To visualize the selected markers with Kaplan–Meier (KM) plots, we used the queue data of published datasets (https://gdc‐portal.nci.nih.gov/) and stratified the patients according to their DNA methylation level. We then used the log‐rank test to examine whether there was a significant difference in the OS rates between the two patient groups. All statistical analyses were performed using R (v4.1.1).

### 2.9 Patient Enrollment Criteria and Blood Sample Acquisition

The cases were diagnosed using routine pathological diagnostic methods such as tissue sections, immunohistochemistry, and blood tests. A total of five cases of primary lymphoma, eight cases of glioma, three cases of intracranial space‐occupying lesions, two cases of meningioma, one case of neuroepithelial tumor, and three cases of metastatic tumor were identified.

### 2.10 Cell Culture and Treatment

The cell lines used in this study were purchased from the ATCC Cell Bank (https://www.atcc.org/). Methylation is performed using the kit method using the MethylCode bisulfite conversion kit (Thermo Fisher, US). The demethylation treatment was drug treatment, decitabine (MCE, US) was configured at 5 mM/mL, and 2 uL was added to each cell to the final concentration of 5uM. After treatment for 24 h, cell RNA was extracted for use.

### 2.11 qPCR

RNA was extracted from plasma using TRIzol solution. Reverse transcription of mRNA was performed according to the M5 First Strand cDNA Synthesis Kit method (Jumei Company), and RT‐qPCR was performed on the cDNA obtained by reverse transcription. The experimental procedure was performed using a PerfectStartTM Green qPCR SuperMix kit. Primers and their sequences are listed in Table 1.

**TABLE 1:** qPCR primer sequences used in this study.

Primer	Sequence 5′→3′	Length
RHEB‐M‐F	TTTGGGAAGGTTGTGTTTATATTAC	192bp
RHEB‐M‐R	GTCAATTCTAATATCCGCTTCG	
RHEB‐U‐F	TTGGGAAGGTTGTGTTTATATTATG	192bp
RHEB‐U‐R	CATCAATTCTAATATCCACTTCACT	
mRHEB‐F	CGAAGCGGATATTAGAATTGAC	438bp
mRHEB‐R	GAATAAACTAACTCCTCCCTCGAA	

## 3 Results

### 3.1 Analysis of Methylation Sites

**FIGURE 1:**
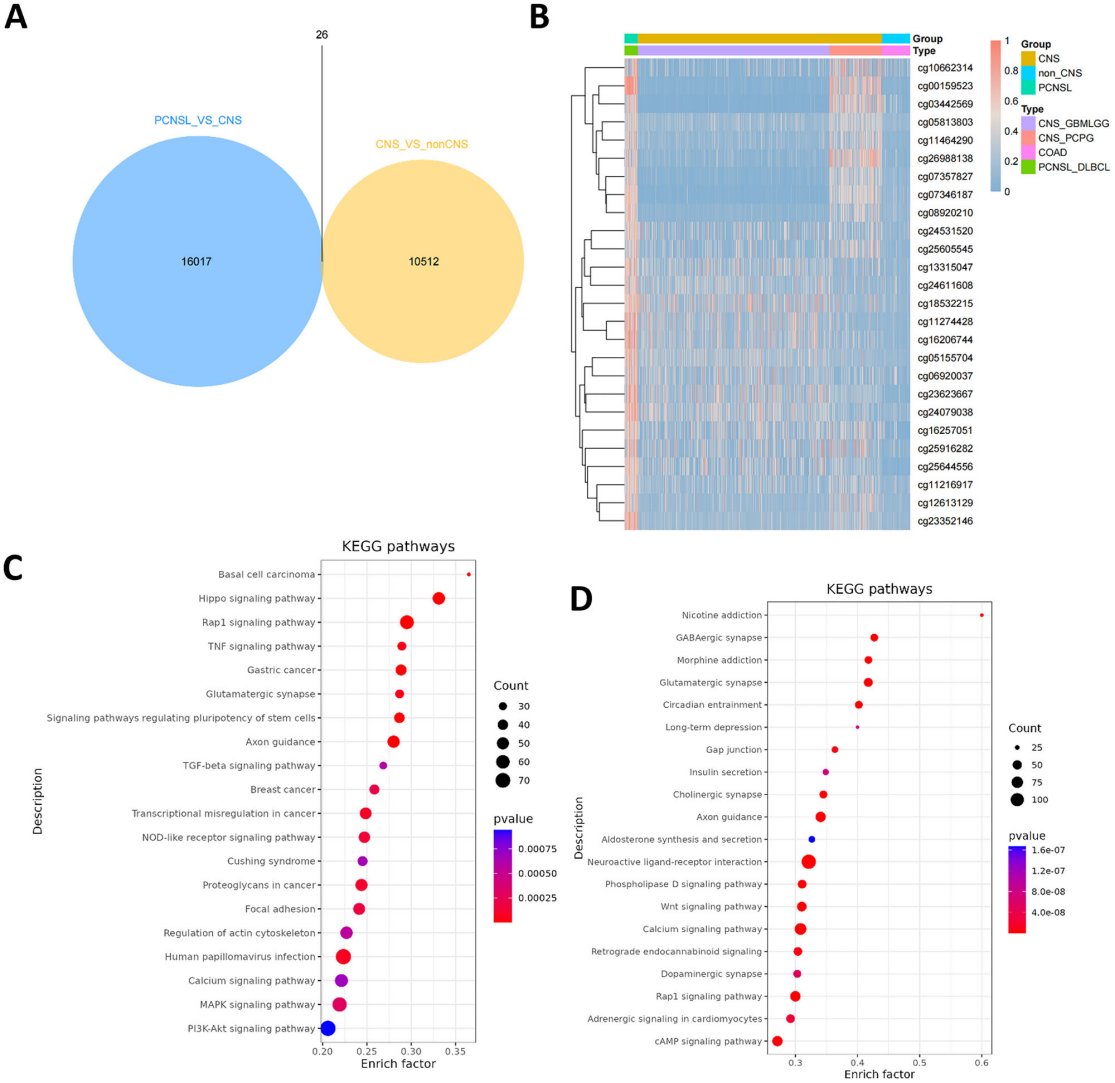
Differential methylation sites of PCNSL and CNS diseases analyzed at 450K. (A) Venn diagram showing differential CpG methylation overlap between PCNSL versus CNS (16,043) and non‐CNS tumors (10,538) (450K) (26 sites). (B) Heatmap of the overlap 26 sites among PCNSL, CNS, non‐CNS tumors (450K). (C) KEGG enrichment of CNS_VS_nonCNS difference sites. (D) KEGG enrichment of PCNSL_vs_CNS difference sites.

The differential methylation sites of the CNS and PCNSL, CNS and non‐CNS groups in the downloaded data were analyzed using the 450 K chip. The results showed 16,043 differential methylation sites in the PCNSL versus CNS group. There were 10,538 differential methylation sites in the CNS versus non‐CNS group and 26 overlapping sites, indicating that a total of 26 differential methylation sites in CNS and PCNSL disease were obtained by 450 K data analysis. Figure 1B shows a thermal map of the expression differences of 26 differentially methylated sites in CNS and PCNSL. Methylation levels at these 26 sites were higher in the PCNSL group, whereas their expression levels in the CNS and non‐CNS groups were significantly lower than those in the PCNSL group. Further enrichment analysis of CNS versus non‐CNS and PCNSL versus CNS difference sites is shown in Figure 1C,D. The enrichment results of CNS_VS_ non‐CNS difference sites showed that the different sites were mainly concentrated in human papillomavirus infection. A comprehensive comparison of the pathway information before the two subgroups showed that pathways with a large number of genes, such as human papillomavirus infection and the PI3K‐Akt signaling pathway, were significantly enriched in both subgroups.

### 3.2 RRBS Analysis of Methylation Sites

**FIGURE 2:**
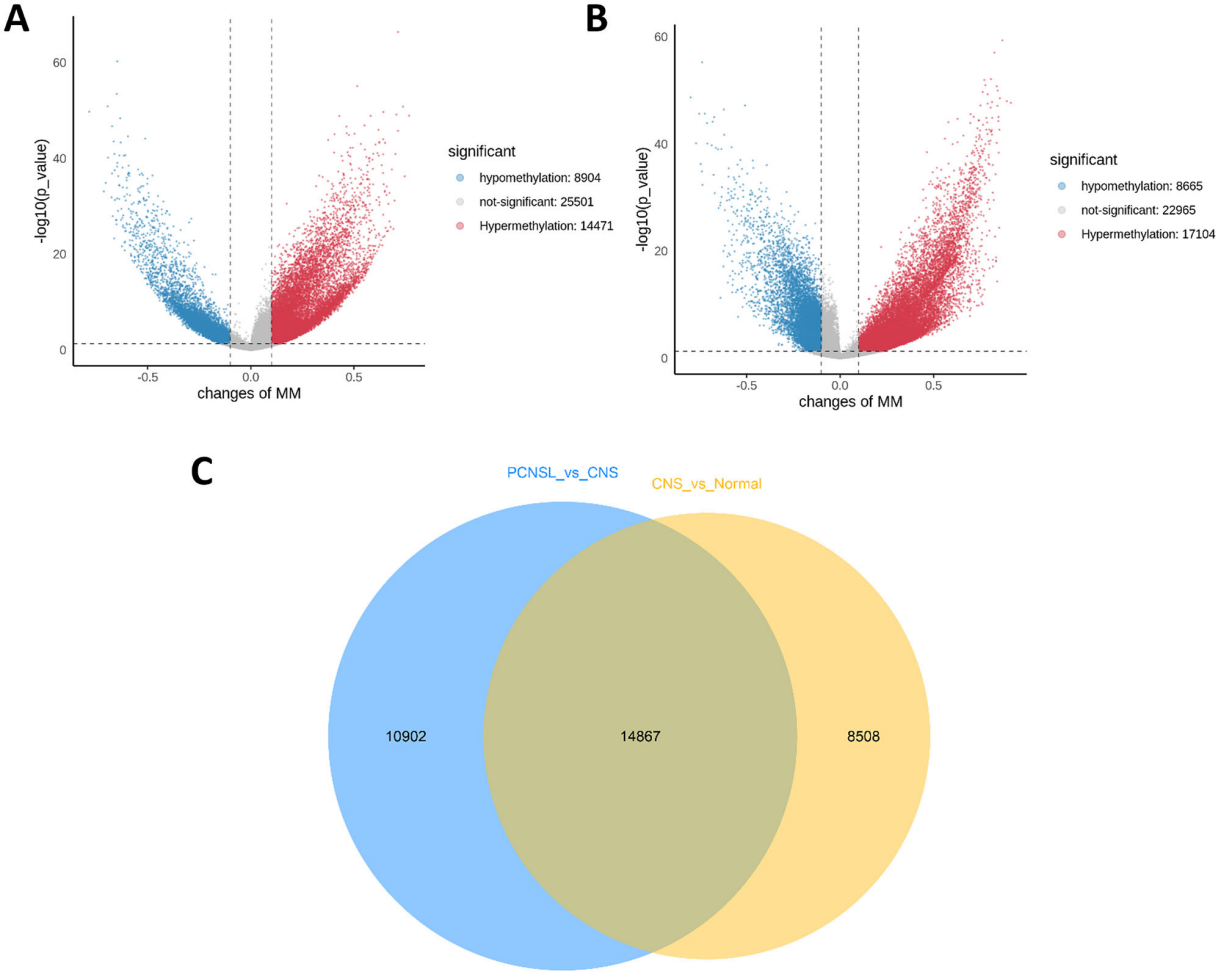
RRBS analysis of differential methylation sites in PCNSL and CNS diseases. (A) Volcano plot of different RRBS methylation sites in CNS versus non‐CNS tumors (8094 hypo‐, 14,471 hyper‐methylated). (B) Volcano plot of different RRBS methylation sites in PCNSL versus CNS (8665 hypo‐, 17,104 hyper‐methylated). (C) Venn diagram showing different RRBS methylation sites overlap between PCNSL versus CNS and non‐CNS tumors (14,867 sites).

RRBS was used to analyze the differential methylation sites in the CNS, normal, CNS, and PCNSL groups in the downloaded data; the results are shown in Figure 2. The normal versus CNS differential volcano map showed 14,471 sites with significantly upregulated methylation in the CNS group. There were 8904 downregulated methylation sites. The CNS versus PCNSL differential volcano map showed 17,104 sites of significant up‐methylation and 8665 sites of down‐methylation in the PCNSL group. The above differential expression sites were analyzed using a Venn diagram between the two comparisons, and the results showed that there were 14,867 overlapping methylation sites, indicating that there were 14,867 common differential methylation sites in the two differential groups analyzed using RRBS data; that is, 14,867 disease differential methylation sites in the CNS and PCNSL were obtained.

### 3.3 Marker Selection of Methylation Gene

**FIGURE 3:**
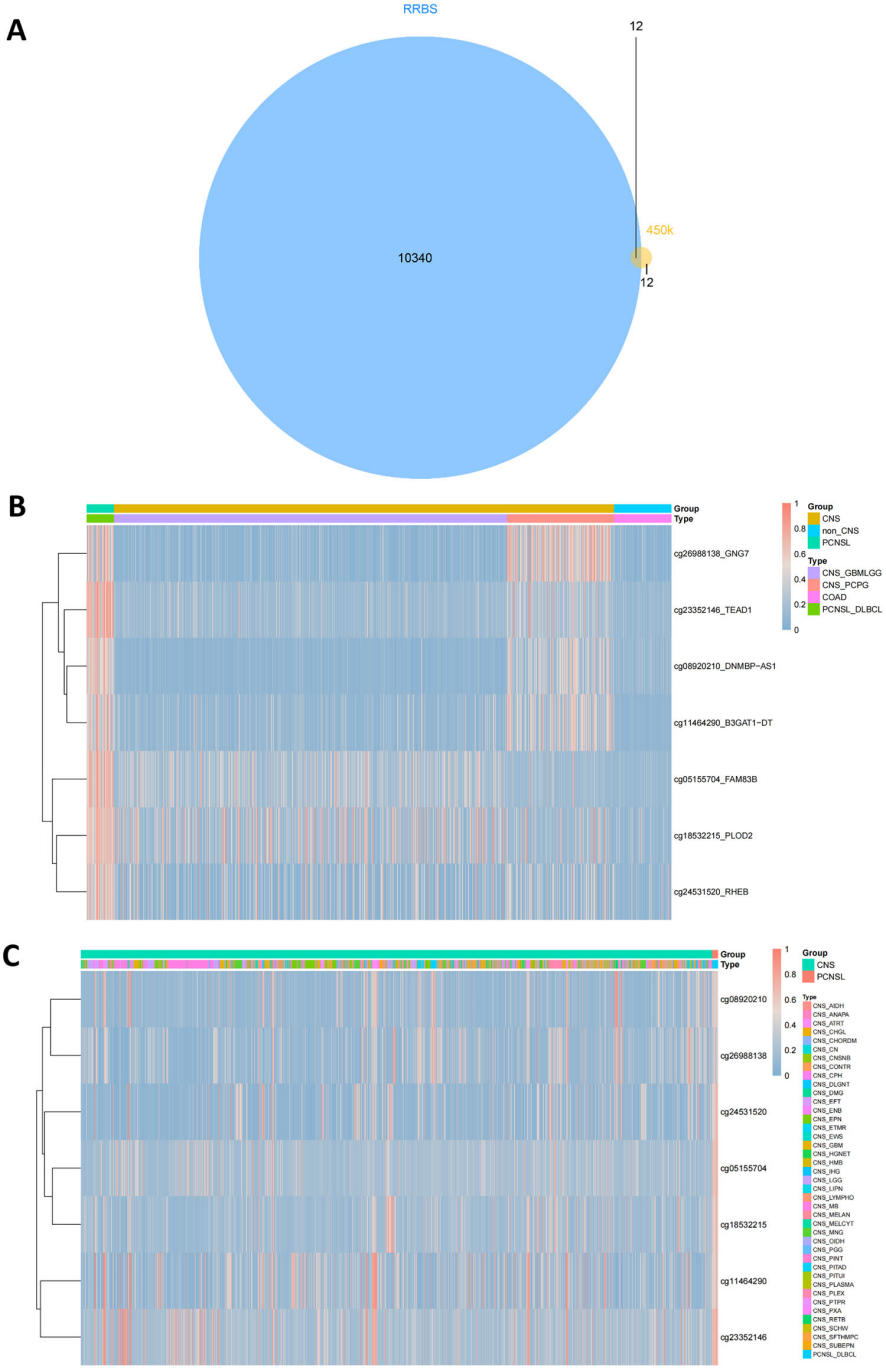
Sets of differential methylation sites in PCNSL and CNS diseases obtained by the two analysis methods. (A) Venn diagram showing overlap between 450K (26 sites) and RRBS (14,867 sites) (12 candidates). (B) Heatmap of methylation levels for seven overlapping candidate genes across PCNSL, CNS, and non‐CNS tumors. (C) Heatmap of methylation levels for seven overlapping candidate genes validated by GEO datasets.

Intersection analysis was performed on the 26 differentially methylated sites obtained from the 450 K analysis and the 14,867 differential methylation sites obtained from the RRBS analysis. As shown in Figure 3A, 12 marker genes in the 450 K chip were shared with the RRBS differentially methylated genes. After removing the CpG site from the distal intergene region, seven methylation sites are associated with the gene promoter or CDS region. Heat map analysis was conducted by comparing the disease data of PCNSL and CNS, the results are shown in Figure 3B. The seven selected markers showed significantly higher expression in the PCNSL group than in the other CNS groups. Pan‐tumor analysis of the CpG marker sites and 82 solid tumors showed significant classification results.

### 3.4 Curve Analysis of Methylation Marker Gene

**FIGURE 4:**
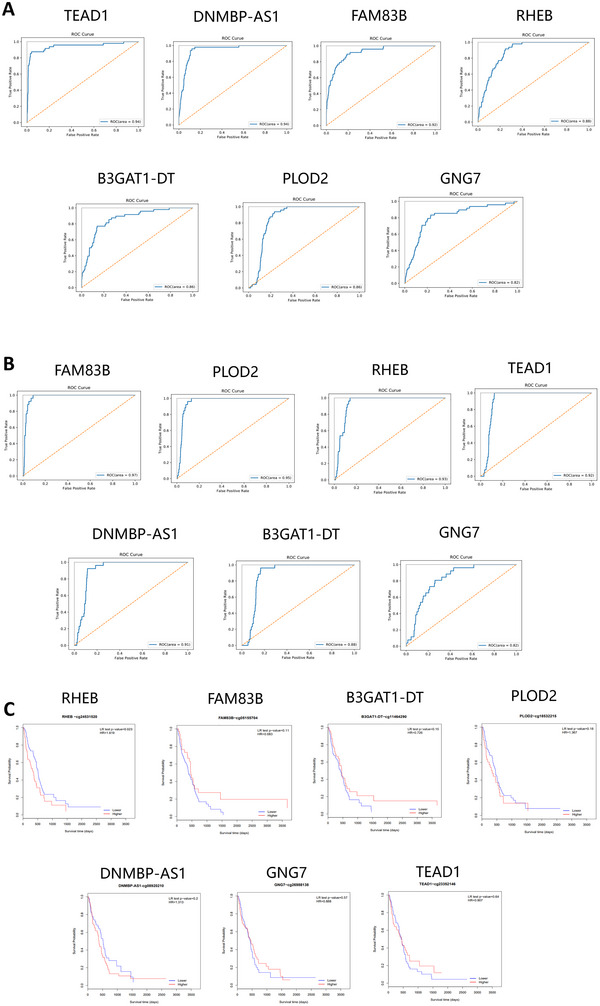
ROC analysis of candidate genes; RHEB promoter methylation showed the highest diagnostic performance. ROC curves analyzed from (A) TCGA database; (B) GEO datasets; and (C) Kaplan‐Meier analysis of overall survival.

Receiver operating characteristic (ROC) curve analysis of marker genes was performed using TCGA data, and the results are shown in Figure 4A. The marker gene had a positive effect on the classification model of CNS and PCNSL based on TCGA data, and the reliability was high, with ROC values above 0.8. At the same time, GEO data were used for ROC curve analysis, and the results are shown in Figure 4B. It was highly reliable the ROC values were both above 0.8.

Survival curves for the seven marker genes were analyzed, and the results are shown in Figure 4C. RHEB, FAM83B, B3GAT1‐DT, PLOD2, DNMBP‐AS1, GNG7, and TEAD1 were associated with poor prognosis, and there was no significant prognostic difference between the high‐ and low‐expression groups.

### 3.5 Clinical Sample Testing

**FIGURE 5:**
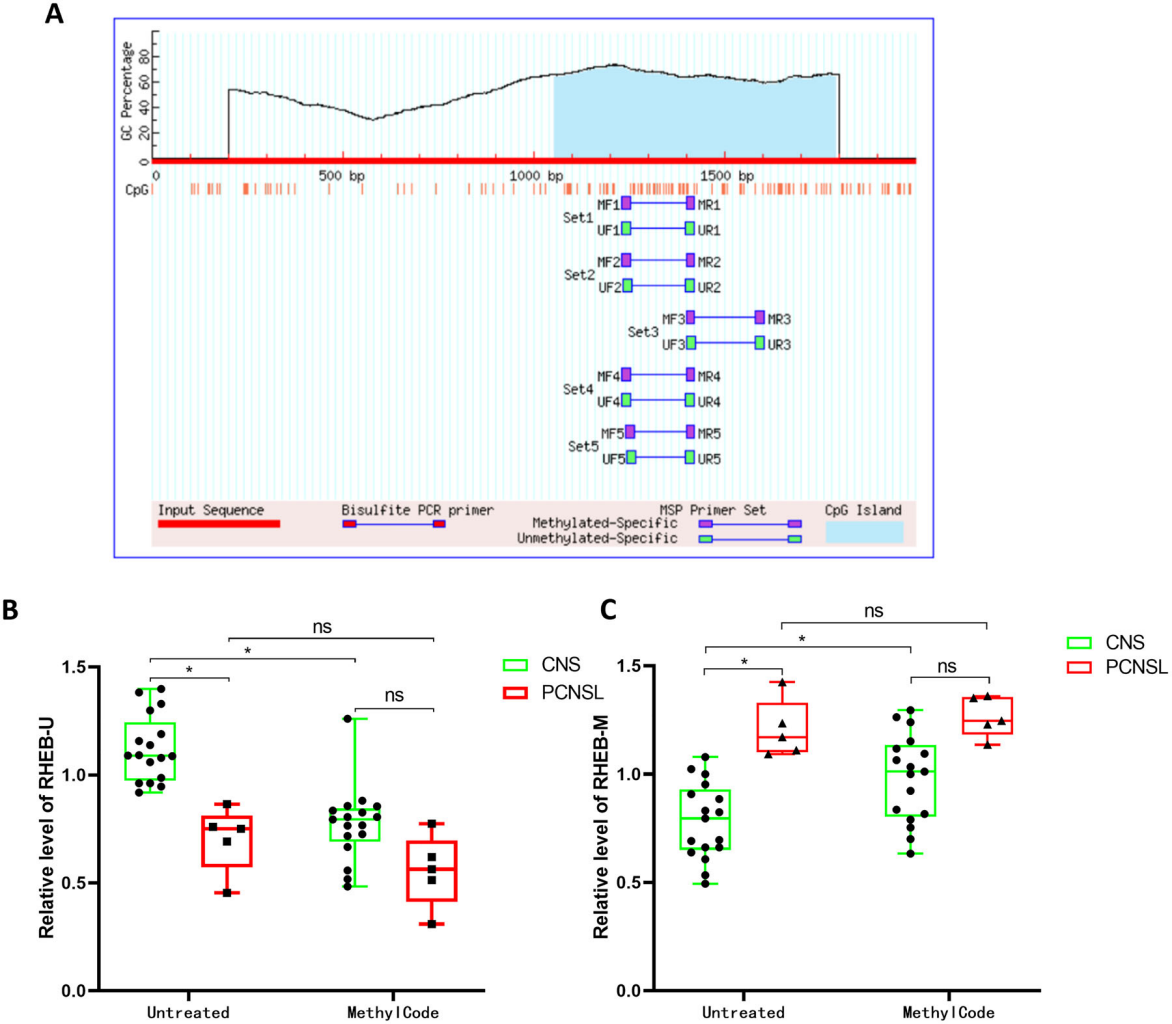
Plasma validation of RHEB promoter methylation. (A) R RHEB gene promoter methylation site primer design (there was only one CpG island in the promoter region of RHEB. We selected a pair of methylating primers, RHEB‐M, and a pair of non‐methylating primers in the same region, RHEB‐U). (B, C) Boxplot of RHEB promoter methylation (B: RHEB‐U; C: RHEB‐M) levels in plasma from PCNSL (*n* = 5) and other intracranial tumors (CNS, *n* = 17).

To validate these results, we selected the best‐performing RHEB genes for follow‐up studies. As shown in Figure 5A, there was only one CpG island in the promoter region of RHEB. We selected a pair of methylating primers, RHEB‐M, and a pair of non‐methylating primers in the same region, RHEB‐U, and used 22 clinical samples to explore whether the designed primers could distinguish the methylation levels of gene promoter regions in CNS and PCNSL patients, as shown in Figure 5B,C. RHEB‐U primer qPCR results showed that compared with CNS patient samples, the expression of RHEB‐U in PCNSL patient samples was significantly down‐regulated, and there was no significant difference between the expression of RHEB‐U in PCNSL patient samples and CNS patient samples after the additional application of pro‐methylation drugs. Compared to before and after treatment with methylated drugs, the expression of the RHEB‐U region in patients with CNS decreased significantly, while the expression of the RHEB‐U region in patients with PCNSL did not change significantly. The results also indicated that RHEB was highly methylated in PCNSL tumors, and the methylation level of RHEB treated with methylating drugs did not change.

### 3.6 Cell Methylation and Demethylation

**FIGURE 6:**
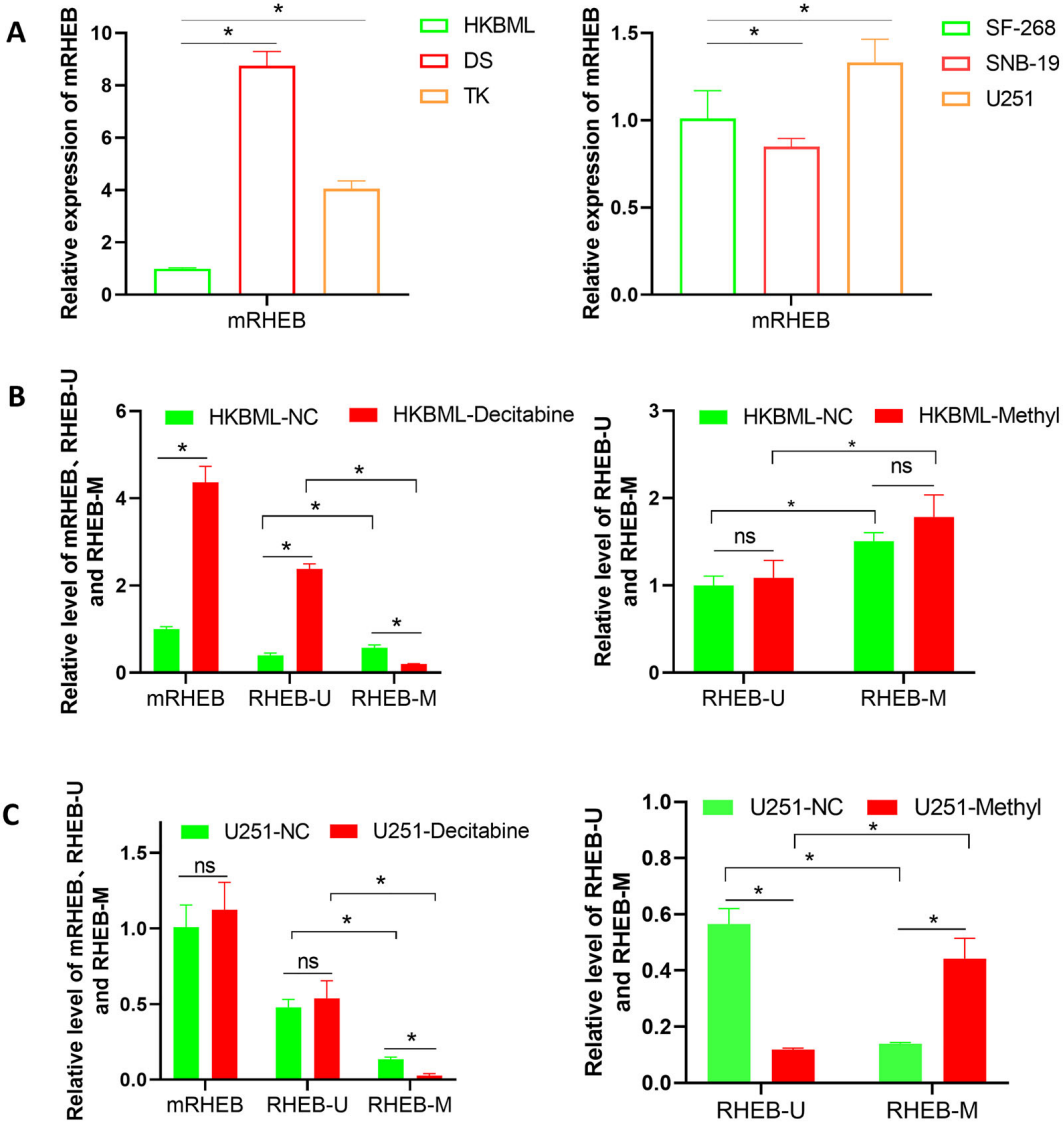
Functional regulation of RHEB by promoter methylation in cell lines. (A) PCNSL and CNS tumor cell screening of RHEB expression. (B) The expression levels of CDS region primers, methylated region universal primers and methylated region methylated primers in PCNSL cancer cells before and after HKBML drug treatment were detected. (C) Detection of CDS region primers, methylated region universal primers and methylated region methylated primers expression levels in CNS cancer cells before and after drug treatment of U251.

We measured expression levels in the CDS regions of HKBML, DS, and TK PCNSL cells and SF‐268, SNB‐19, and U251 CNS cells. The results showed that the HKBML cell line had the lowest expression of RHEB among the PCNSL cell lines, the DS cell line had the highest expression, the SNB‐19 cell line had the lowest expression of RHEB among the CNS cell lines, and the U251 cell line had the highest expression (Figure 6A).

PCNSL cell lines with low expression levels and the CNS tumor cell line U251 with high expression levels were selected for methylation and demethylation drug treatment, and the results are shown in Figure 6B,C. In HKBML cells, before demethylation treatment, qPCR results of the CDS region primer (mRHEB), unmethylated region primer (RHEB‐U), and methylated region primer (RHEB‐M) of RHEB showed that the expression and methylation of this gene were all low. Compared with mRHEB, Both RHEB‐U and RHEB‐M were lower, and RHEB‐U was significantly lower than RHEB‐M. After treatment with demethylated drugs, the results of mRHEB and RHEB‐U primers increased significantly, whereas the results of RHEB‐M primers decreased significantly compared to those before treatment.

In U251 cells, the trend in the detection results of the three primers before and after demethylation treatment was the same as that in HKBML cells. However, unlike in HKBML cells, the expression levels detected by mRHEB and RHEB‐U primers did not change significantly after demethylation treatment and did not show any increase. In U251 cells, the methylation level of RHEB‐M significantly increased after methylation treatment, but the unmethylated level of RHEB‐U significantly decreased.

## 4 Discussion

PCNSL is an extranodal non‐Hodgkin lymphoma with great clinical differences and is difficult to diagnose. Routine histopathology and immunohistochemistry remain the gold standards for the diagnosis and classification of PCNSL. Currently, there are a few studies on the development of molecular markers with low specificity (Gonzalez‐Gomez et al. 2003; Chu et al. 2006). Therefore, we developed markers that can be used for the diagnosis of PCNSL based on DNA methylation. To obtain PCNSL‐specific molecular markers extensively and accurately, two analytical methods–450 K and RRBS–were used to analyze the data. The Illumina HumanMethylation450 BeadChip (450 K, Illumina) contained 485,512 probes covering 99% of the RefSeq genes. The 450k array is an extension of the earlier BeadChip, the HumanMethylation27 array (27k array). The dynamic ranges of the two probe types are slightly different, which may lead to type II bias during the analysis (Wang et al. 2019). Although not as comprehensive as sequence‐based methods, it is more affordable and easier to analyze and interpret. These properties make 450k arrays ideal for epigenome‐wide association studies (EWAS) involving hundreds or even thousands of cohort samples (Bhat and Jones 2022; Wei et al. 2021; Lucas et al. 2019), and can also be used to identify methylation signatures as biomarkers of disease status and progression (Zhou et al. 2022; Hannon et al. 2021). Reduced‐representation bisulfite sequencing (RRBS) is an analytical method for detecting biological methylation that was developed more than a decade ago (Ziller et al. 2011) and has been widely used since its initial development (Guo et al. 2014; Klughammer et al. 2018; Schrott et al. 2020; Stryjewska et al. 2017; Szymczak et al. 2020). This new method simplifies and improves the analytical workflow, enabling hundreds of MSC‐RRBS libraries to be formed within 2 days (Charlton et al. 2018; Gaiti et al. 2019). For example, single‐cell sulfite sequencing (scBS‐Seq) (Smallwood et al. 2014; Clark et al. 2017) and single‐core methyllysine sequencing (snmC‐Seq) (Luo et al. 2018) use random primers with indexed tails to replicate sulfite‐transformed single‐stranded DNA. In this study, 12 methylation sites with significant differences were obtained using two analysis methods. It was verified that the methylation levels of these sites in tumor patients could distinguish PCNSL patients, indicating that these sites have research value for further verifying the development of primers for clinical use in subsequent studies. Among the methylation sites, only one in the RHEB gene was located in the promoter region, whereas the other methylation sites were located in the CDS region. We selected patient tumor tissues and performed in vitro cell experiments to verify the differentiation and sensitivity of RHEB methylation primers to PCNSL patients through methylation level detection. In other words, a pair of PCNSL‐specific primers may be developed for clinical plasma detection, but its performance still needs to be tested in more clinical samples.

Despite the strengths of combining large‐scale bioinformatic analyses with clinical and experimental validation, several limitations should be acknowledged. First, the size of our clinical cohort was small, which limits the statistical power of our findings and underscores the need for validation in larger and independent patient populations. Second, although we cross‐validated our findings using multiple publicly available datasets (TCGA, GEO, CCLE), external replication in multicenter cohorts with diverse clinical and ethnic backgrounds remains essential. Third, our reliance on RHEB promoter methylation as a single diagnostic marker constrains translational potential. Future studies should evaluate additional methylation markers identified in this study (e.g., FAM83B, B3GAT1‐DT, PLOD2, DNMBP‐AS1, GNG7, TEAD1) and explore their integration into a multi‐marker panel to improve diagnostic sensitivity and specificity. Together, these steps will be critical to advance our findings from preliminary, proof‐of‐concept observations toward clinical application.

## 5 Conclusion

The methylation difference in the RHEB gene promoter region shows promise as a distinguishing feature between the CNS diseases and PCNSL. The primer sequence designed in this study represents a proof‐of‐concept molecular marker. However, these findings are preliminary and require validation in larger and independent clinical cohorts before translation into a clinically applicable diagnostic tool.

## Author Contributions

**Yan Feng**: conceptualization, formal analysis, methodology, visualization, writing–original draft, writing–review and editing. **Wang Yaming**: conceptualization, formal analysis, methodology, visualization, writing–original draft, writing–review and editing. **Fan Xiaotong**: data curation, methodology, validation. **Wei Penghu**: data curation, methodology, validation. **Shan Yongzhi**: conceptualization, data curation, writing–review and editing.

## Ethics Statement

This study protocol was reviewed and approved by Ethics Committees of Xuanwu Hospital Capital Medical University (Approval No. LYS [2021]118).

## Consent

Written informed consent was obtained from the participants prior to the study.

## Conflicts of Interest

The authors declare no conflicts of interest.

## Peer Review

The peer review history for this article is available at https://publons.com/publon/10.1002/brb3.70996.

## Data Availability

The datasets used and/or analyzed during the current study are available from the corresponding author on reasonable request.
